# Congenital Obstruction of the Colon in an Infant

**Published:** 1847-05

**Authors:** James Bryan


*Congenital obstruction of the Colon in an infant.* By JAMES BRYAN, M. D.

*April 2d*, 1847.—On the 31st ult., I was requested by my friend Dr. L. P. Gebhard to make a post obit examination of J. B., a male infant three days old, who had died without having had any discharge from its bowels after birth. The child had been cheerful and took the breast at first, but gradually became languid, and sunk without evident cause, except the want of a discharge per anum.

On inspection of the body, the abdomen was found very much enlarged and of a greenish colour. The capillaries of the skin were injected, and the cuticle of a purple colour, as low as half way down the thighs, involving the genital organs, groins, &c.; the abdomen was tympanitic; the os frontis was freely separated down to the root of the nose; the general appearance of the infant in other respects was that of health.

On making an incision through the integuments in the direction of the linea alba, a large quantity of fœtid gas escaped from the abdominal cavity. The bladder appeared healthy and contained a little urine; the uracus was attached to the umbilicus and bladder; the peritoneum in every direction was engorged with dark coloured blood, and the circulatory arcades of the mesocolon with the small vessels of the colon were filled with blood, and easily seen when the intestine was held between the eye and the light. The greater part of the hypogastric region was filled by a tumour about four inches in diameter. On opening the colon above this tumour (which proved to be situated in the sigmoid flexure of the colon,) large quantities of meconium and fœcal matter were discharged. The peritoneum when divided was found to cover a smooth surface of the tumour; this surface proved to be the outer coat of the intestine, which was very much distended and contained the tumour in the form of coagulated blood, as black as pitch. The descending colon was distinctly traced into the covering of the tumour, and was found to adhere to the margin of the pelvis, and to be entirely *imperforate* in the direction of the rectum.

The anus was in a natural condition; a probe would pass without difficulty, and had been passed during life up the rectum to the tumour or obstruction. A few drops of blood had followed the introduction of the probe. The genital organs were well developed; so also the liver, stomach, and small intestines. The tissue of the intestines around the tumour was found *apoplexied*.

The explanation of this case would appear to be, that a rupture of some of the vessels of the sigmoid flexure of the colon took place during uterine life. A gradual discharge of blood into the intestine and between its coats took place, producing pressure on the surrounding parts, and in this way, inducing adhesions to an extent to close the passage of the blood towards the rectum, and causing the general adhesions which were found outside of the intestine.

The *treatment* of this case, *provided the diagnosis had been made out*, would be, to make an opening into the tumour through the rectum; this failing on account of the adhesions, we might perhaps resort to the production of an artificial anus in one of the situations usually recommended ; in this case the latter operation would probably have relieved the patient, at least temporarily.

---

To the Editor of the Medical Examiner.

*Sir*,—Permit me, through the pages of your journal, to direct the attention of the younger members of the profession to a practice, which prevails more or less with many of them, of asking for farther advice in difficult cases of their seniors, by letter, and thus subjecting them to manifest inconvenience and responsibility, without accompanying their request with the wonted, or any, *honorarium*. Where special relations exist, or have existed, as between preceptor and pupil, there may be apparent, if not real, propriety in the latter seeking occasionally for farther enlightenment ; but wherever the patient can afford to rèmunerate the consulting physician, it ought most assuredly to be the pleasure—if not the duty—of the attending physician to suggest the appropriate accompaniment. The undersigned has been subjected to repeated inconvenience on this head, and he knows that others have been equally annoyed.

*Philadelphia*, *April* 17, 1847. IATROS.

[We publish the foregoing communication with pleasure, in the hope that it may be useful in correcting an evil of which we hear many and just complaints. We often receive communications complaining, indeed, of eminent physicians for not having answered letters asking counsel, and invariably we have found that the letters sent contained no fee, and sometimes were not even postage paid.—EDITOR.]

